# A DTC morphometrics package for quantification of complex and variable cellular morphology using ImageJ

**DOI:** 10.17912/micropub.biology.001202

**Published:** 2024-05-20

**Authors:** Nilay Gupta, Michael Cammer, Theadora Tolkin, E. Jane Albert Hubbard

**Affiliations:** 1 Department of Cell Biology, NYU Grossman School of Medicine, New York, New York, United States; 2 Department of Biology, New York University, New York, New York, United States; 3 Microscopy Laboratory, Division of Advanced Research Technology, NYU Grossman School of Medicine, New York, New York, United States

## Abstract

Quantification of complex cellular morphology is important for understanding developmental control of cell shape as well as the developmental ramifications of dysregulated cell shape. However, processing and scoring 3D confocal micrographs can be time consuming and prone to errors such as sample-data matching for large datasets, reproducibility between users, and errors introduced by variable image quality. These problems are further compounded where cell shapes vary from sample to sample and intensity dynamic ranges extend over orders of magnitude. Here we present a package of ImageJ macros we developed for analysis of the *C. elegans* hermaphrodite distal tip cell (DTC) to (a) optimize images for analysis and (b) assist in quantifying various features of the cell by two independent methods, one user-guided and the other unbiased. Together these tools provide functionality for visualization and multiple parameters of quantification which can be easily customized within free open-source ImageJ.

**Figure 1. Overview of image processing and markup.**
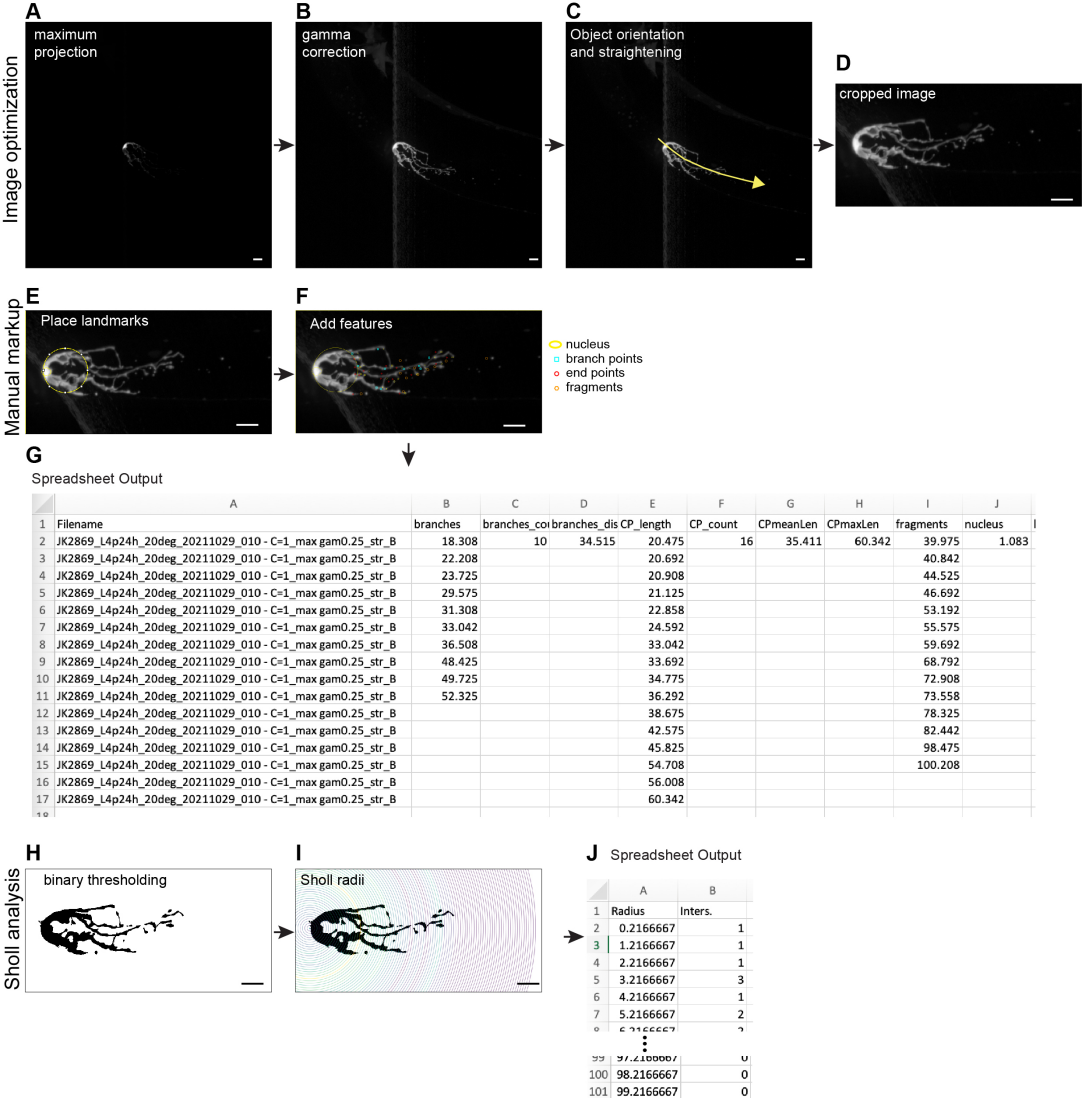
A) A maximum projection image of an Z-confocal series (file format .nd2), with no contrast adjustment or filtering. B) Maximum projection image created from the same image file, using “Max project and change contrast” command. C) Same panel as in B, with yellow segmented line showing path used in “Rotate and straighten based on curved line” command. The arrow indicates the direction the line must be drawn to ensure the orientation is correct (distal to the left) when rotated. D) Final rotated, straightened, cropped image. E) Cropped image with the large circle positioned tangent to the curve of the distal end of the gonad and the smaller circle placed around the center of the nucleus. Both circles are placed manually by user. F) Same image as in E, with features manually curated by user. G) Screenshot of spreadsheet output that results from “Measure all markups” command. H) Binary image that results from “Prepare for Sholl plugin” command. Note that for visual clarity, the contrast in this image has been inverted from the actual result in ImageJ. I) Same image as in H, with Sholl radii shown every 1µm. J) Screenshot of spreadsheet output from the Neuroanatomy plugin that runs the Sholl analysis (Ferreira et al. 2014). The screenshot has been trimmed to show the number of intersections that occur in the first 7 and final 3 radii. Worms imaged bear the *qIs57* [ *lag-2p* ::GFP] transgene (Seigfried et al., 2004; Davis et al., 2022). Scale bar is 10 µm in all images.

## Description

The *C. elegans* hermaphrodite distal tip cell (DTC) is the unicellular niche for germline stem cells. The DTC controls the fate of stem cells through expression of the DSL ligands *lag-2* and *apx-1* , which bind and activate the Notch/GLP-1 receptor on the germline stem cells (GSCs) in a contact-dependent manner. In adults, the DTC forms an elaborate plexus of processes that grow around and intercalate between dozens of germline stem cells. This cell is a powerful system for cell-biological studies because of its dynamic morphological changes throughout development and because of its role as a stem cell niche (Kimble and White, 1981; Henderson et al., 1994; Nadarajan et al., 2009; Byrd et al., 2014; Linden et al., 2017; Kocsisova et al., 2019; Urman et al., 2024) . Nevertheless, to understand developmental changes in DTC morphology and the relevance DTC morphology to germline stem cells, it is necessary to establish a robust set of quantifiable parameters that can be used to capture differences in DTC morphology under different experimental conditions.

Quantitative analysis of DTC morphology can be performed using 3-D rendering software such as Imaris (e.g., Linden et al., 2017). A major benefit of this approach is that many parameters are captured in well-segmented cells, including unforeseen parameters that researchers may wish to examine post-hoc without re-imaging. However, use of Imaris imposes software licensing costs and the availability of sufficient computer power to run the software. In addition, we found obtaining images of sufficient quality to perform accurate segmenting of the DTC was not always possible. In particular, accurate segmentation can prove problematic in circumstances where tangential but irrelevant fluorescence exists in the sample that cannot be separated from the object of interest (e.g. gut granules versus gonad in live *C. elegans* ).

To maximize throughput of reproducible quantitative measurements of the DTC while minimizing time and effort, we needed a means to generate images that captured DTC-specific fluorescence – even across orders of magnitude of intensity – and a means to perform and log measurements associated with each individual specimen directly in ImageJ/Fiji. The package presented here includes three general components: image optimization, DTC manual markup, and bitmap image creation. The first component includes a single step to create a maximum projection image and adjust contrast to maximize visualization, even if the fluorescent dynamic range covers orders of magnitude, and a step to rotate and straighten the image. The second component allows users to create a manual markup of up to seven distinct cell morphology parameters, a command to log all parameters associated with a single image and to save that data as a tab-delimited text file with a unique automatic filename assignment that matches the specimen filename. The third component reduces the number of steps required to use thresholding to create a binary image suitable for Sholl analysis, which is an established method for quantifying complex neuronal morphology, and which can be applied to quantifying DTC morphology, using the SNT plugin (Ferreira et al., 2014) .

Although the macros were designed for DTC measurements, we envisage that, with minimal modification, they could be applied to quantify parameters for other cell types that exhibit variable morphology.

**Overview of the macro components**

Here, we provide a general overview of each of the macro components and an example of its use. A detailed step-by-step user manual and test images are provided as Extended Data.

Image optimization: This component of the macro starts from a 0.5 µm Z-series through a fluorescently marked DTC ( Fig. 1A ). The first part of the macro collects a maximum projection, performs contrast adjustment via gamma transformation, and automatically subtracts background based on the darkest pixels of the image (Fig 1B). These steps ensure that the contrast of all images is in the same range. The user then uses the curved line drawing function native to ImageJ to position the DTC (distal left, proximal right) and to straighten it. To generate images of uniform dimensions, the package crops the image using a fixed box size ( Fig. 1D ). The entire image is then copied to the ROI manager to provide backup for lost images and for later error-checking as necessary. In addition to providing images for analysis as described below, the images at this point can be made into montages that are useful for comparing multiple variable cells, using ImageJ tools.

DTC manual markup: Based on varying features we observed among montages of multiple DTCs imaged with different fluorescent reporters, we established a versatile slate of morphological features of the DTC for mark-up (e.g., branchpoints, puncta, fragments, process endpoints), and commands to assist the user in mark-up to record the number and position (along the distal-proximal axis) of each feature. To start, the user designates the distal end of the gonad as the intersection of the distal end of the gonad with the inner arc of a circle. Subsequent marks are logged according to their distance along the X-axis from that point. A second, smaller circle marks the position of the nucleus, a feature visible with only certain markers, but that can change position with age (Kocsisova et al., 2019; Urman et al., 2024) . A marked image and the corresponding macro output is shown in Figure 1 . To ensure that the macro output was robust, we compared DTC markups from five relatively inexperienced users, and, with one round of subsequent guidance, the results were consistent across users.

Sholl analysis:

An alternative method for capturing general complexity without the need for manual markup is Sholl analysis (Sholl, 1953). This method has been used extensively in measurements of neurons and a separate package has been created for obtaining neuronal morphometry directly from bitmap images (Ferreira et al., 2014) . We have applied that package here to the DTC ( Figure 1E -G). In short, the method generates radii at a specified spacing around a user-defined centerpoint. The number of intersections of each radius (in this case 1 µm apart) with pixels above the threshold is recorded. We included a step in our package to create the bitmap image necessary for Sholl analysis with the SNT package.

## Methods

*Image acquisition*

The macros require input which is a high-resolution single channel low noise Z stack capturing very dim (thin processes) to very bright (cell body) signal over orders of magnitude without saturation or clipping. Subsequent contrast enhancement is designed for wide dynamic range low noise images. We chose spinning disk microscopy because it is more than 10X faster than laser scanning confocal and captured details that were lost in scattering by deconvolution or Zeiss Apotome. Selective plane illumination microscopy image capture (SPIM) may be effective, but we have not tested it.

Z stacks were collected at 0.5 µm steps with a Nikon W1 spinning disk confocal microscope with an Apo 60x 1.40 Oil λS DIC N2 0.13 WD lens, Andor 888 Live EMCCD camera with 10 MHz readout, conversion gain 1, and low EM gain for low noise 0.2167 um pixels. To image GFP, a 488 nm laser was used with a 89101 quad 405/488/555/640 dichroic and 525/50 filter.

Laser power was in the in 2% to 25% range with exposure times in 50 to 200 ms range for maximum pixel intensity between 10,000 and 50,000. With a low noise camera of this type, there is no need for the brightest pixels to exceed 10,000 when gain settings are low. If using a laser scanning confocal, it may need to be set at 16 bits with photon counting accumulation set to fill a substantial dynamic range without saturation or averaging with a traditional photomultiplier tube.

See additional notes regarding specific confocal setups in the Extended Data manual.

Three test image stacks (.nd2 files) are provided in the Extended Data.

*Image optimization, DTC manual markup, Sholl analysis steps*

See Extended Data for user manual and test image stacks.

## Extended Data

Description: Macro file. Resource Type: Text. DOI: 10.22002/ya1gg-j4484

Description: User Manual. Resource Type: Text. DOI: 10.22002/g04bh-7br78

Description: Test image stack (1 of 3). Resource Type: Image. DOI: 10.22002/2jka3-p5j06

Description: Test image stack (2 of 3). Resource Type: Image. DOI: 10.22002/dqy9v-q7s06

Description: Test image stack (3 of 3). Resource Type: Image. DOI: 10.22002/7bk32-03963

## References

[R1] Davis P, Zarowiecki M, Arnaboldi V, Becerra A, Cain S, Chan J, Chen WJ, Cho J, da Veiga Beltrame E, Diamantakis S, Gao S, Grigoriadis D, Grove CA, Harris TW, Kishore R, Le T, Lee RYN, Luypaert M, Müller HM, Nakamura C, Nuin P, Paulini M, Quinton-Tulloch M, Raciti D, Rodgers FH, Russell M, Schindelman G, Singh A, Stickland T, Van Auken K, Wang Q, Williams G, Wright AJ, Yook K, Berriman M, Howe KL, Schedl T, Stein L, Sternberg PW (2022). WormBase in 2022-data, processes, and tools for analyzing Caenorhabditis elegans.. Genetics.

[R2] Ferreira TA, Blackman AV, Oyrer J, Jayabal S, Chung AJ, Watt AJ, Sjöström PJ, van Meyel DJ (2014). Neuronal morphometry directly from bitmap images.. Nat Methods.

[R3] Henderson ST, Gao D, Lambie EJ, Kimble J (1994). lag-2 may encode a signaling ligand for the GLP-1 and LIN-12 receptors of C. elegans.. Development.

[R4] Kimble JE, White JG (1981). On the control of germ cell development in Caenorhabditis elegans.. Dev Biol.

[R5] Kocsisova Z, Kornfeld K, Schedl T (2019). Rapid population-wide declines in stem cell number and activity during reproductive aging in C. elegans.. Development.

[R6] Linden LM, Gordon KL, Pani AM, Payne SG, Garde A, Burkholder D, Chi Q, Goldstein B, Sherwood DR (2017). Identification of regulators of germ stem cell enwrapment by its niche in C. elegans.. Dev Biol.

[R7] Nadarajan S, Govindan JA, McGovern M, Hubbard EJ, Greenstein D (2009). MSP and GLP-1/Notch signaling coordinately regulate actomyosin-dependent cytoplasmic streaming and oocyte growth in C. elegans.. Development.

[R8] SHOLL DA (1953). Dendritic organization in the neurons of the visual and motor cortices of the cat.. J Anat.

[R9] Siegfried KR, Kidd AR 3rd, Chesney MA, Kimble J (2004). The sys-1 and sys-3 genes cooperate with Wnt signaling to establish the proximal-distal axis of the Caenorhabditis elegans gonad.. Genetics.

[R10] Urman MA, John NS, Jung T, Lee C (2024). Aging disrupts spatiotemporal regulation of germline stem cells and niche integrity.. Biol Open.

